# Oxidative Stress Contributes to Slit Diaphragm Defects Caused by Disruption of Endocytosis

**DOI:** 10.1016/j.ekir.2023.11.018

**Published:** 2023-11-25

**Authors:** Gang Xi, Sajan A. Lamba, Michael Mysh, John S. Poulton

**Affiliations:** 1 UNC Kidney Center, Department of Medicine, University of North Carolina at Chapel Hill, Chapel Hill, North Carolina, USA

**Keywords:** endocytosis, oxidative stress, podocyte, protein trafficking, reactive oxygen species, slit diaphragm

## Abstract

**Introduction:**

Podocyte slit diaphragms are an important component of the glomerular filtration barrier. Podocyte injury frequently includes defects in slit diaphragms, and various mechanisms for these defects have been described, including altered endocytic trafficking of slit diaphragm proteins or oxidative stress. However, the potential relationship between endocytosis and oxidative stress in the context of slit diaphragm integrity has not been extensively considered.

**Methods:**

To examine the potential relationships between endocytosis, oxidative stress, and slit diaphragm integrity, we induced genetic or pharmacological disruption of endocytosis in *Drosophila* nephrocytes (the insect orthologue of podocytes) and cultured human podocytes. We then employed immunofluorescence microscopy to analyze protein localization and levels, and to quantify signal from reactive oxygen species (ROS) dyes. Immunoprecipitation from podocyte cell lysates was used to examine effects on slit diaphragm protein complex formation (i.e., nephrin/podocin and nephrin/ZO-1).

**Results:**

Disruption of endocytosis in nephrocytes and podocytes led to slit diaphragm defects, elevated levels of ROS (oxidative stress), and activation of the nuclear factor erythroid 2–related factor 2 (Nrf2) antioxidant pathway. In nephrocytes with defective endocytosis, perturbation of Nrf2 signaling exacerbated slit diaphragm defects. Conversely, overexpression of Nrf2 target genes catalase or glucose-6-phosphate dehydrogenase (G6PD) significantly ameliorated slit diaphragm defects caused by disruption of endocytosis.

**Conclusion:**

Oxidative stress is an important consequence of defective endocytosis and contributes to the defects in slit diaphragm integrity associated with disruption of endocytic trafficking.

## Graphical abstract

**Figure:**
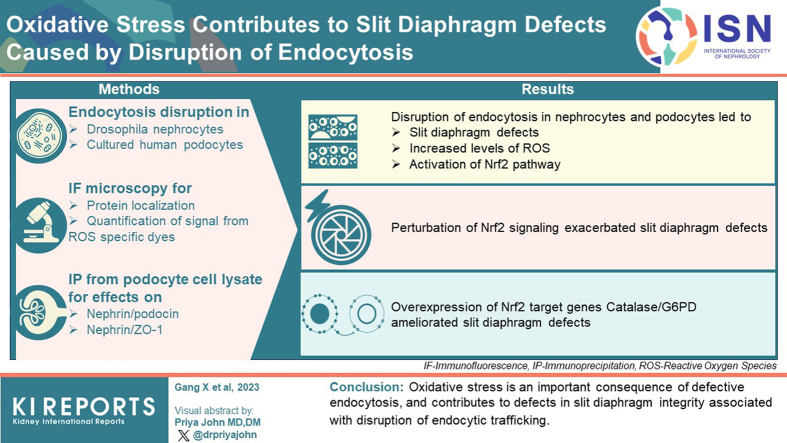

Podocyte slit diaphragms contribute to size-selective glomerular filtration and are disrupted in many forms of kidney disease. Slit diaphragm defects can lead to proteinuria and podocyte foot process effacement. Molecularly, slit diaphragms are a highly specialized cell-cell junction comprised of a unique suite of adhesion molecules and cytoskeletal adapters. The transmembrane proteins nephrin and neph1 are core components of the slit diaphragm,^1^^,^^2^ and studies indicate that regulated endocytic trafficking of these proteins is critical for maintaining the structure and signaling capabilities of the slit diaphragm.^3^, ^4^, ^5^, ^6^, ^7^, ^8^, ^9^, ^10^, ^11^ Studies of *Drosophila* nephrocyte slit diaphragms, a highly conserved model of podocyte slit diaphragms, also demonstrate that proper endocytic trafficking is critical for nephrin turnover and slit diaphragm integrity.^12^, ^13^, ^14^, ^15^ These reports highlight the direct effects of endocytic trafficking on slit diaphragms proteins, and consequently the integrity of the slit diaphragm. However, endocytic trafficking is a fundamental cellular process affecting countless aspects of cell behavior; therefore, our understanding of the myriad ways in which defective protein trafficking may impact slit diaphragms is likely incomplete.

In addition to proper protein trafficking, slit diaphragms rely on the podocyte’s ability to maintain cellular redox balance. Many studies of kidney disease in humans or animal podocyte injury models have reported oxidative stress (increased ROS), with potential effects on slit diaphragms.^16^, ^17^, ^18^, ^19^, ^20^, ^21^ For example, rodent studies have shown that reducing oxidative stress by increasing antioxidant levels or depleting major sources of ROS (e.g., NOX4) can ameliorate slit diaphragm defects resulting from various forms of podocyte injury (e.g., diabetes, puromycin, or ischemia).^22^, ^23^, ^24^, ^25^ In addition, some nephrotic syndrome patients bear mutations in enzymes in the coenzyme Q10 biosynthesis pathway.^26^^,^^27^ Coenzyme Q10 is an important mitochondrial antioxidant. Studies in fly nephrocytes demonstrate that coenzyme Q10 deficiency results in oxidative stress and slit diaphragm defects.^28^^,^^29^ Those studies went on to show that antioxidant supplementation significantly reduced slit diaphragm defects, suggesting that elevated ROS can damage slit diaphragms.

Given the pervasiveness of oxidative stress in kidney disease, and its potential effects on slit diaphragms, we hypothesized that oxidative stress may contribute to slit diaphragm defects associated with defective endocytic trafficking. To test this, we disrupted endocytosis in fly nephrocytes and cultured human podocytes; and observed the consequences on redox homeostasis and slit diaphragm integrity. As expected, disruption of endocytosis led to slit diaphragm defects; however, we also observed evidence of oxidative stress and activation of the Nrf2 antioxidant response pathway in both nephrocytes and podocytes. We also found that a significant portion of the slit diaphragm defects caused by loss of major endocytic regulators could be prevented by overexpression of the antioxidant-promoting enzymes, G6PD or catalase; both known downstream targets of Nrf2 signaling.^30^^,^^31^ Together, our findings indicate that, in addition to its known role in trafficking slit diaphragm proteins, endocytosis also promotes slit diaphragm integrity indirectly by promoting redox balance in these cells.

## Methods

### *Drosophila* Genetics and Microscopy

Flies were maintained at 25 °C. Flies of the indicated genotypes were crossed and allowed to lay eggs on standard fly food (Archon Scientific D2 glucose media). Fly stocks used included the following: Dot-Gal4 a gift from Dr. Deborah Kimbrell, University of California, Davis; Sns-Gal4 a gift from Dr. Susan Abmayr, Stowers Institute; Pros-Gal4 - Bloomington 80572; Discs large (Dlg) RNAi - Bloomington 33671; Rab5 RNAi - Bloomington 34832; amnionless (Amn) RNAi - Bloomington 41956; cubilin (Cubn) RNAi - Bloomington 28702; GstD1>GFP a gift from Dr. Dirk Bohmann, University of Rochester^32^; Nrf2 RNAi (fly Cnc) - Bloomington 25984; G6PD RNAi - Vienna Drosophila Resource Center 101507; G6PD overexpression lines a gift from Dr. William Orr, Southern Methodist University; Catalase overexpression - Bloomington 24621; and yellow white (yw; control) - Bloomington 1495. In the fly experiments, control animals were generated by crossing the indicated Gal4 line to yw.

For immunostaining, wandering third instar larvae were dissected and prepared as previously described.^12^ Mouse anti-ZO-1 antibody was used at 1:500 (Developmental Studies Hyrbridoma Bank; anti-fly Polychaetoid, clone PYD2). Alexafluor conjugated antimouse secondary antibodies were used at 1:500 (Invitrogen). Nuclei were labeled with 4′,6-diamidino-2-phenylindole (Sigma). Confocal images were acquired on Zeiss LSM700 and 710 confocal microscopes at the UNC Microscopy Services Laboratory. Quantification of ZO-1 surface localization was performed as follows.^12^ Using ImageJ, we selected a cross-sectional z-slice that was representative of the degree of ZO-1 localization for that cell, as determined by assessing all z-slices of the cell. We then measured the perimeter of the cell in that slice, as well as the total length of that perimeter that was positively enriched for ZO-1. We then divided the ZO-1 positive length by the total perimeter of that cell to determine the percent of the cell surface that is positive for ZO-1. Multiple cells analyzed from the same animal were then averaged (number of cells analyzed per fly ranged from 1–4). Statistical comparisons were performed in GraphPad Prism 9; specific tests applied are indicated in the main text or figure legends. Graphical model images generated in BioRender.com.

For GstD>GFP measurements in the RNAi backgrounds, we created a fly stock containing GstD>GFP (second chromosome) and Pros-Gal4 (third chromosome), and outcrossed those flies to the indicated RNAi or control flies. Wandering third instar larvae were processed for imaging as described above. Images for control and treated animals were acquired on the same day using the same acquisition settings to allow comparison of GFP signal intensity. Because Pros-Gal4 crossed with Dlg RNAi proved to be lethal, we used Dot-Gal4 as the driver for the Dlg knockdown experiment. To analyze GstD>GFP expression following treatment with bardoxolone methyl (MedChemExpress; #HY-13324), the drug was incorporated into fly food (Genessee Nutri-Fly) to a final concentration of 200 μM. GstD>GFP larvae were reared on this food for 6 days until they reached third instar stage (control flies were raised on Nutri-Fly without bardoxolone).

For ROS detection by dihydroethidium (DHE) staining, larvae were dissected in room temperature PBS, then incubated for 5 minutes in 1 ml PBS supplemented with DHE (Invitrogen; Cat# D11347) at 30 μM final concentration. Samples were then washed 3 times for 1 minute each in 1 ml PBS. Samples were then fixed in 4% PFA for 5 minutes, rinsed once with PBS, and mounted for immediate imaging by confocal microscopy. Because DHE binds DNA and fluoresces upon oxidation by ROS, we measured nuclear DHE signal in ImageJ (mean gray value) and standardized that to the cytoplasmic background of the cell. We then analyzed the data using the average of the individual cells measured from the same fly (number of cells/fly ranged from 1–6).

### Human Podocyte Culturing

Conditionally immortalized human podocytes (a gift from Dr. Moin A. Saleem, University of Bristol) were cultured as described previously.^33^ The cells were cultured at 33 °C to allow proliferation until 85% to 90% confluency. The cells were then shifted to 37 °C, allowing the podocytes to differentiate for 10 to 16 days. The expression of podocyte markers (nephrin and podocin) were verified by immunostaining. Mycoplasma tests (4′,6-diamidino-2-phenylindole staining) were regularly performed to ensure cell line purity.

### Immunoprecipitation and Immunoblotting

The cell monolayers were lysed in a modified radioimmunoprecipitation assay buffer. Coimmunoprecipitation was performed by incubating 0.5 mg of cell lysate protein with 1 μg of an anti-nephrin antibody (SC-377246, Santa Cruz) at 4 °C overnight. Immunoblotting was performed using a 1:1000 dilution for anti-ZO-1 (Cat# 61-7300, Invitrogen) and anti-podocin antibodies (P0372, Sigma-Millipore). To control for protein input, cell lysates that contained the same protein amounts were loaded and immunoblotted with a dilution of 1:500 anti-nephrin (AF4269, R&D Systems) or 1:200 anti-α-tubulin antibody (SC-8035, Santa Cruz). The proteins were visualized using enhanced chemiluminescence (ThermoFisher Scientific, Waltham, MA). Total cellular protein in the lysates was determined using BCA (ThermoFisher Scientific).

### ROS Detection in Cultured Podocytes

Podocytes (4 × 10^4^ cells/well) were seeded in a 24-well glass-bottom plate (Cat# EK-42892, E&K Scientific). The differentiated podocytes were treated with indicated amounts of dynasore (Cat#324410, Calbiochem) for 3 hours and washed twice with PBS. DHE (10 μM final concentration in PBS) was loaded into the wells and incubated for 15 minutes. The cells were washed 3 times with PBS before reading the fluorescence using a TECAN plate reader (Infinite M200 PRO) (excitation wavelength 545 nm/ Emission wavelength 605 nm). For the DHE assay with dynasore and catalase treatment, the cells were seeded in a μ-Slide 4 well plate (Ibidi, Cat# 80426), grown at 33 ^o^C, and differentiated at 37 ^o^C. The differentiated podocytes were exposed to dynasore (150 μM) in the presence or absence of catalase (0.5 μM final concentration). After DHE labelling, the cells were covered with mounting medium containing 4′,6-diamidino-2-phenylindole (VECTASHIELD, Vector laboratories, Inc., Newark, CA). Images were captured by an EVOS fluorescent microscope (ThermoFisher Scientific).

## Results

### Endocytosis is Important for Slit Diaphragm Integrity and Redox Balance in Nephrocytes

**Figure 1:**
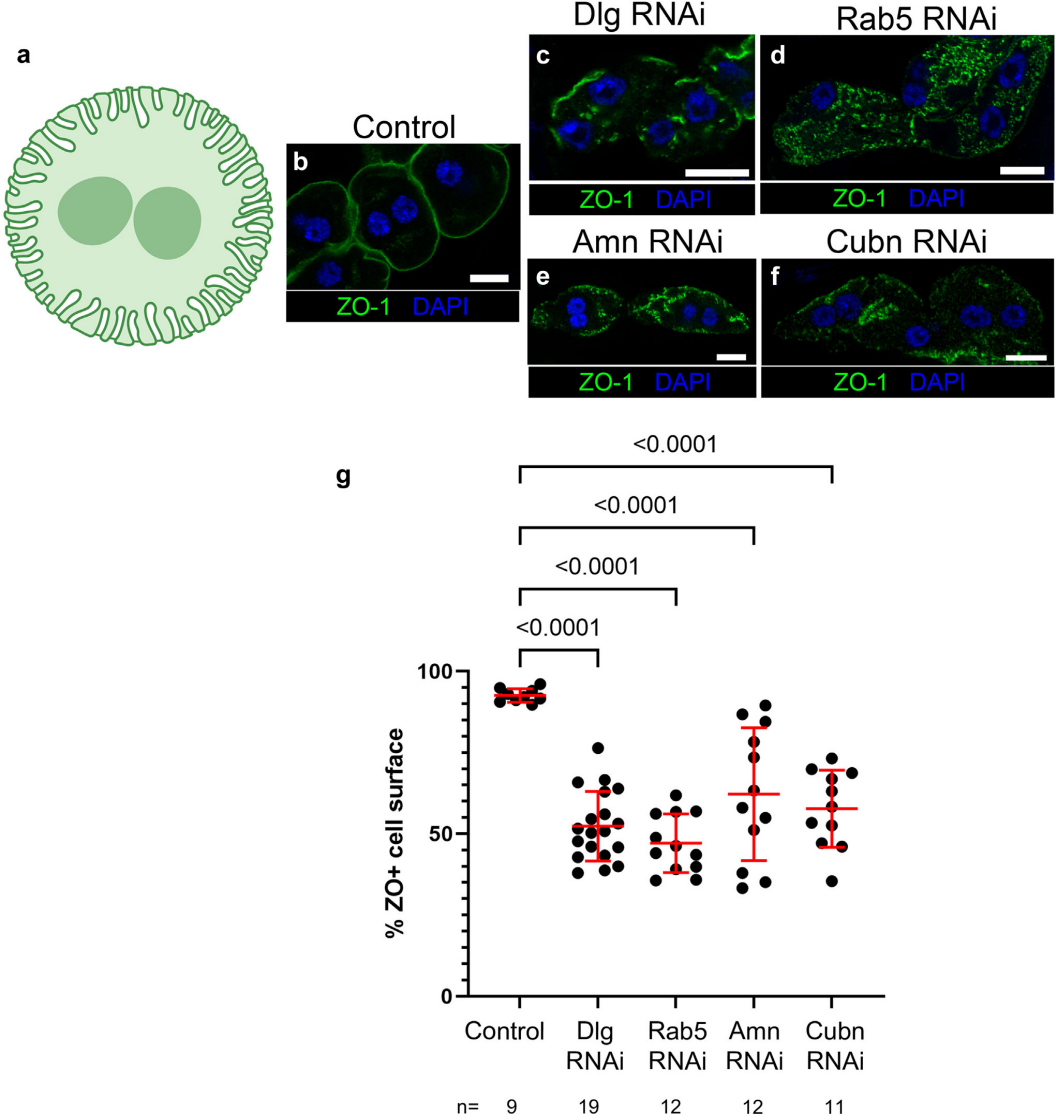
Disruption of endocytosis induces slit diaphragm defects in *Drosophila* nephrocytes. (a) Diagram of nephrocyte architecture. Slit diaphragms are located all over the surface of normal nephrocytes and help seal off the numerous invaginations of the plasma membrane. (b) Cross-sectional image of control nephrocytes (*Sns-Gal4 × yw*) stained with the slit diaphragm protein ZO-1 (green). (c–f) *Sns-Gal4* driving UAS transgenes expressing RNAi hairpins targeting the indicated genes leads to significant loss of ZO-1 from the cell surface and into cytoplasmic aggregates. (g) Quantification of ZO-1 loss from the cell surface. All RNAi knockdown nephrocytes are significantly different from control by 1-way analysis of variance with Dunnett’s multiple comparisons test. Each data point represents the average of the nephrocytes scored from an individual fly. “*n*” indicates the number of flies analyzed. Scale bars = 10 microns.

To examine the potential relationship between endocytosis, oxidative stress, and slit diaphragm integrity, we first took advantage of the fly nephrocyte, a well-established model of vertebrate podocytes that form molecularly and functionally conserved slit diaphragms. One notable distinction is that podocytes form slit diaphragms between adjacent podocytes, whereas nephrocyte slit diaphragms are intracellular junctions on the surface of a single cell, where they seal off numerous projections of the plasma membrane (Figure 1a). In doing so, they create compartments of extracellular space known as labyrinthine channels. Importantly, the plasma membrane lining these channels conducts endocytosis at very high rates. This combination of filtration at the slit diaphragm and endocytosis in the labyrinthine channels allow nephrocytes to perform their function of filtering the fly blood (hemolymph).^34^, ^35^, ^36^

To perturb endocytic trafficking in nephrocytes, we depleted these cells of previously described regulators of endocytosis—Dlg, Rab5, Cubn, and Amn. Dlg is an important factor in nephrocyte endocytosis and slit diaphragm integrity.^12^^,^^37^ Rab5 (a key regulator of early endocytic vesicle formation) is a major mediator of nephrocyte slit diaphragm integrity and trafficking of slit diaphragm proteins.^13^^,^^14^ The Cubn-Amn complex mediates protein uptake in renal proximal tubule cells, and is also critical to nephrocyte function.^38^, ^39^, ^40^ It has been noted that the labyrinthine channels of fly nephrocytes, with their high levels of endocytosis and proximity to the slit diaphragm filtration barrier, bear functional similarity to proximal tubule cells of the vertebrate nephron.^40^

To examine the potential role of oxidative stress in mediating slit diaphragm defects caused by disruption of endocytic trafficking, we first confirmed that depletion of these endocytic regulators (Dlg, Rab5, Cubn, or Amn) led to disruption of slit diaphragm formation. Using the Gal4-UAS system to drive RNAi hairpins in nephrocytes, we observed significant mislocalization of the slit diaphragm protein ZO-1 from the cell surface and into ectopic intracellular aggregates (Figure 1b–g). We and others have previously demonstrated that ZO-1 specifically localizes to the slit diaphragm in fly nephrocytes, and its mislocalization from the cell surface is a reliable indicator of slit diaphragm disruption.^12^^,^^41^, ^42^, ^43^

**Figure 2:**
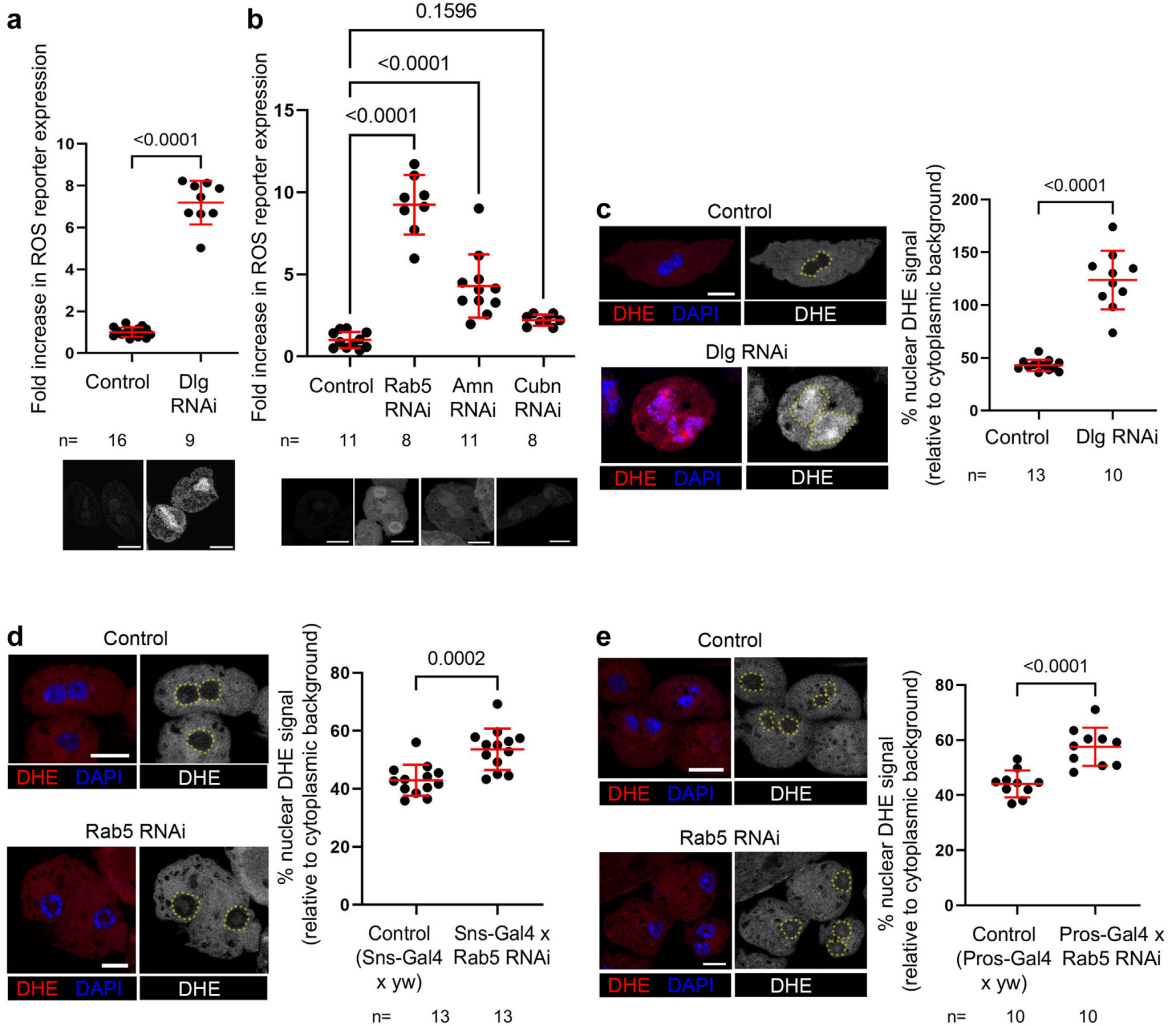
Disruption of endocytosis leads to oxidative stress in fly nephrocytes. (a) Nephrocytes depleted of Dlg using Dot-Gal4 have significantly increased expression of the ROS reporter GstD>GFP, compared to controls. Expression levels normalized to the mean of the control and analyzed by Welch's unpaired t-test. (b) Nephrocytes depleted of Rab5 or Amn using Pros-Gal4 also show significantly increased ROS reporter expression. Data normalized to control mean and analyzed by analysis of variance with Dunnett's correction for multiple comparisons. Representative gray-scale images of GFP channel are shown below indicated genotypes for each graph (image acquisition settings held constant and postacquisition changes applied equally to all treatments and controls). (c) Dlg knockdown nephrocytes have significantly increased nuclear DHE signal (red), relative to controls; Welch's unpaired t-test. Nuclei in gray scale images are demarcated by yellow dotted lines. (d) Similarly, knockdown of Rab5 also significantly elevates nuclear DHE staining, though the overall effect is modest. (e) Knockdown of Rab5 using a different Gal4 driver (Pros-Gal4) indicates a similar modest but significant increase in ROS levels. DAPI labeling of nuclei in blue. Scale bars = 10 microns. Amn, amnionless; DAPI, 4′,6-diamidino-2-phenylindole; DHE, dihydroethidium; Dlg, discs large; ROS, reactive oxygen species.

We then determined if disruption of endocytosis also affected redox balance in nephrocytes. To do so, we knocked down Dlg, Rab5, Cubn, or Amn, and examined ROS levels using the genetically encoded ROS-reporter, GstD>GFP.^32^ This reporter contains the *gstD1* enhancer region, which includes a Nrf2 binding site known as the antioxidant response element. As described below, Nrf2 protein levels are positively regulated by ROS, and Nrf2 in turn regulates expression of many antioxidant genes via binding to antioxidant response element sites. Thus, as ROS levels rise, so do Nrf2 levels, and consequently so do Nrf2 target genes, such as GstD1. To confirm that this reporter reflects Nrf2 activity, we treated flies with the Nrf2 activator, bardoxolone methyl. Indeed, larvae reared on food containing bardoxolone methyl showed dramatic upregulation of GstD>GFP in multiple tissues and cell types, demonstrating its utility as a reporter of Nrf2 activity (Supplementary Figure S1A–F). When we incorporated this reporter into nephrocytes depleted of the endocytic regulators Dlg, Rab5, or Amn, we observed a significant increase in reporter expression, suggesting oxidative stress conditions in these cells (Figure 2a and b). We measured an approximately 2-fold increase in GstD>GFP expression in Cubn RNAi cells; however, it did not reach statistical significance. Because Dlg RNAi crossed with Pros-Gal4 led to early larval lethality, we could not assess Dlg knockdown using the Pros-Gal4 line; instead, we used Dot-Gal4 crossed to Dlg RNAi in that experiment, with Dot-Gal4 crossed to yw as the control (Figure 2a).

To confirm increased ROS levels following knockdown of endocytic regulators, we performed DHE staining in control and Dlg knockdown nephrocytes. DHE is a ROS-sensitive dye that, upon oxidation, binds to DNA and emits red fluorescence.^44^ In agreement with our GstD>GFP reporter data, we observed a substantial increase in nuclear fluorescence in Dlg knockdown nephrocytes, compared to control (Sns-Gal4 crossed with yw) (Figure 2c). DHE staining of nephrocytes expressing Rab5 RNAi under control of Sns-Gal4 indicated a modest but statistically significant increase in ROS levels when compared to control (Figure 2d; Sns-Gal4 crossed with yw). We repeated DHE staining in Rab5 knockdown nephrocytes using a second Gal4 line (Pros-Gal4), which returned very similar results—modest but significant increase in nuclear DHE relative to control (Figure 2e). We also analyzed the effects of depleting Cubn or Amn on DHE signal intensity, but did not detect a significant increase using that assay (Supplementary Figure S1G and H). Together, these data indicate that disruption of endocytosis, through depletion of various endocytic regulators, can lead to both oxidative stress and slit diaphragm defects.

### Oxidative Stress is a Key Contributor to Slit Diaphragm Loss Caused by Defects in Endocytosis

We next wished to determine if oxidative stress in nephrocytes with defective endocytic trafficking is an important factor in the slit diaphragm defects occurring in these cells. To do so, we first considered how cells respond to oxidative stress. A primary cellular response to oxidative stress involves activation of the KEAP1-Nrf2 pathway, a major transcriptional regulator of antioxidant levels.^31^^,^^45^ For cells in redox balance, KEAP1 normally binds to Nrf2 (a transcription factor), which mediates ubiquitination and proteasomal degradation of Nrf2. However, as ROS levels increase, KEAP1 is directly oxidized, which inhibits its ability to bind Nrf2. Nrf2 then accumulates in the cell, translocates to the nucleus, and activates transcription of a host of genes encoding antioxidant and detoxification proteins, including catalase, glutathione S-transferases, and G6PD.^30^^,^^31^^,^^45^

**Figure 3:**
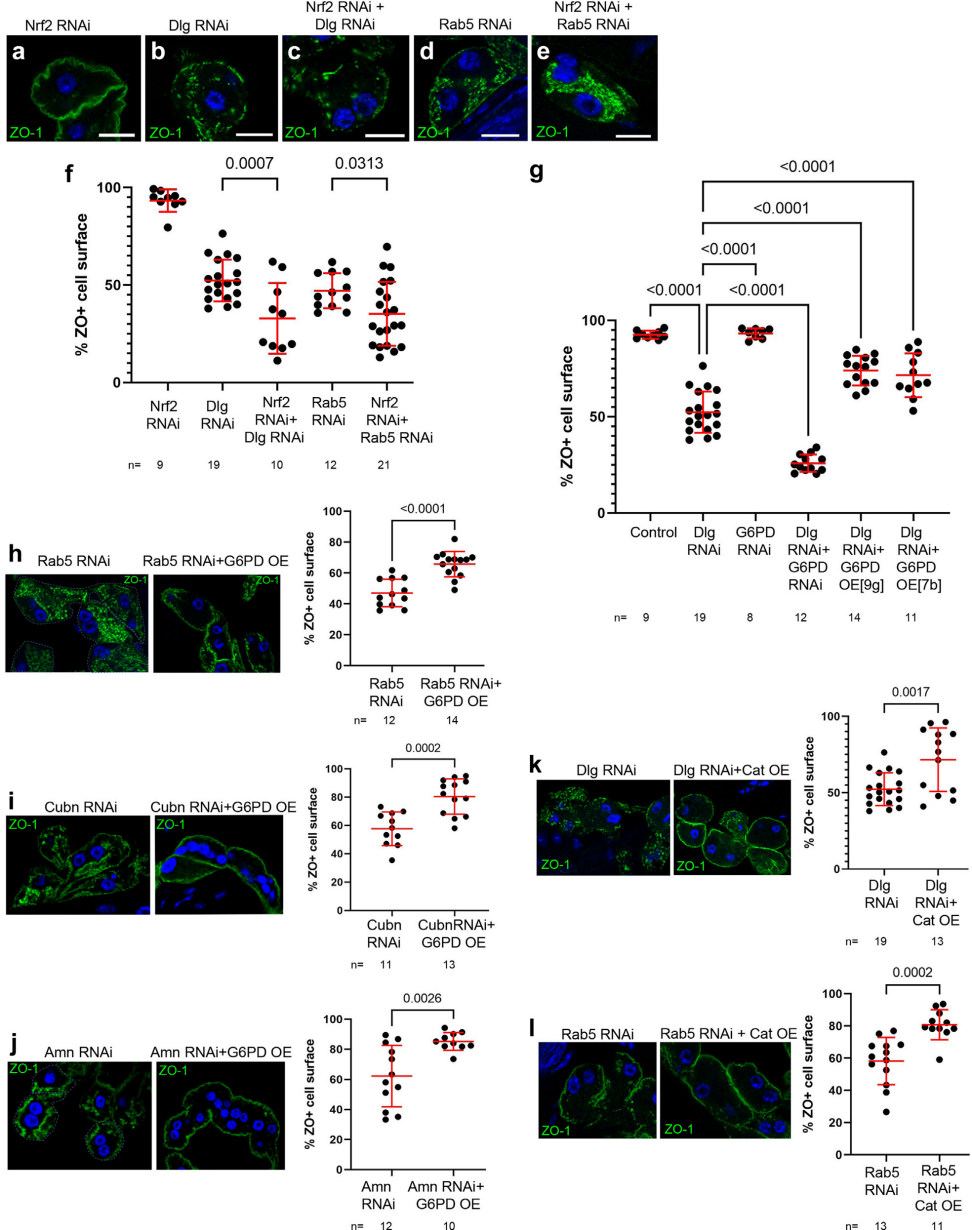
Oxidative stress is an important mediator of slit diaphragm defects caused by disruption of endocytosis. (a) Knockdown of Nrf2 alone does not perturb slit diaphragm integrity, as indicated by normal localization of ZO-1 to the nephrocyte surface. (b) Dlg knockdown significantly disrupts ZO-1 localization. (c) Codepletion of Nrf2 and Dlg results in a dramatic enhancement of slit diaphragm loss, relative to either single knockdown. (d) Rab5 knockdown disrupts ZO-1 localization. (e) Double knockdown of Nrf2 and Rab5 increases the degree of ZO-1 mislocalization. (f) Quantification of ZO-1 surface localization. All genotypes with Dlg or Rab5 knockdown are significantly different from Nrf2 alone, but those *P*-values are not shown for simplicity. (g) Although depletion of G6PD alone does not disrupt slit diaphragms, codepletion of G6PD and Dlg leads to significant mislocalization of ZO-1 from the cell surface. Conversely, overexpression of G6PD in Dlg knockdown nephrocytes significantly rescues ZO-1 localization. Two different transgenic insertions of UAS-G6PD (9g and 7b) were tested, with similar results. (h) The disruption of ZO-1 mislocalization caused by knockdown of Rab5 can be significantly improved by overexpression of G6PD. Overexpression of G6PD led to similar improvements in ZO-1 surface localization in nephrocytes depleted of Cubn or Amn (i and j, respectively). (k) Slit diaphragm disruption caused by Dlg knockdown can also be significantly rescued by overexpression of the antioxidant catalase. (l) Similar results were observed in Rab5 depleted cells with catalase overexpression. Sns-Gal4 was used for the experiments in this figure because it produces moderate defects when depleting cells of these endocytic regulators, which provides sufficient sensitivity in our assay to detect enhancement or suppression of the phenotype. ZO-1 staining in green; DAPI staining of nuclei in blue. Scale bars = 10 microns. Amn, amnionless; Cubn, cubilin; DAPI, 4′,6-diamidino-2-phenylindole; Dlg, Dlg, discs large; G6PD, glucose-6-phosphate dehydrogenase; Nrf2, nuclear factor erythroid 2–related factor 2.

To examine a potential protective role of Nrf2 on nephrocyte slit diaphragms, we first examined ZO-1 localization in cells depleted of Nrf2 alone. We found that reducing Nrf2 in otherwise normal nephrocytes had no detectable effect on slit diaphragm integrity (Figure 3a and f). However, when we challenged nephrocytes with disruption of endocytosis (Dlg RNAi or Rab5 RNAi), and simultaneously depleted them of Nrf2, we observed a reduction in slit diaphragm integrity significantly worse than either RNAi alone (Figure 3b–f). This suggests that Nrf2 helps limit the extent of slit diaphragm loss caused by disruption of endocytosis, which we hypothesize is due to its role in countering oxidative stress by promoting antioxidant expression.

We therefore wished to know if Nrf2 target genes were involved in buffering nephrocytes against the deleterious effects of oxidative stress caused by disruption of endocytosis. We first examined the effects of manipulating expression of G6PD. G6PD is the rate limiting enzyme in the pentose phosphate pathway and a major producer of NADPH.^46^^,^^47^ NADPH is used by glutathione reductase to catalyze the production of reduced glutathione, a potent antioxidant of hydrogen peroxide (H_2_O_2_), a principle form of ROS in most cells.^48^ We tested whether G6PD participates in the cellular response to depletion of Dlg by knocking down Dlg and G6PD, alone and in combination. Similar to our findings with Nrf2 manipulation, loss of G6PD alone had no discernable effect on slit diaphragms, whereas codepletion of Dlg and G6PD significantly enhanced the slit diaphragm defects caused by loss of Dlg (Figure 3g). We then wished to determine if oxidative stress was contributing to the slit diaphragm defects in Dlg knockdown cells. To do this, we overexpressed G6PD in the Dlg RNAi background, and found that increased G6PD levels were sufficient to significantly restore slit diaphragm integrity in Dlg knockdown cells (Figure 3g). We then extended this approach to nephrocytes depleted of the endocytic regulators Rab5, Cubn, or Amn. In each case, we found that the slit diaphragm defects caused by knockdown of the given endocytic regulator could be significantly ameliorated by overexpression of G6PD (Figure 3h–j).

We performed a similar rescue experiment using overexpression of another Nrf2 target, catalase.^31^ Catalase is an antioxidant, able to reduce H_2_O_2_. Similar to our G6PD experiments, we found that increased catalase expression improved slit diaphragm integrity in nephrocytes depleted of Dlg or Rab5 (Figure 3k and l).

### Disrupting Endocytosis Leads to Oxidative Stress in Cultured Human Podocytes

**Figure 4:**
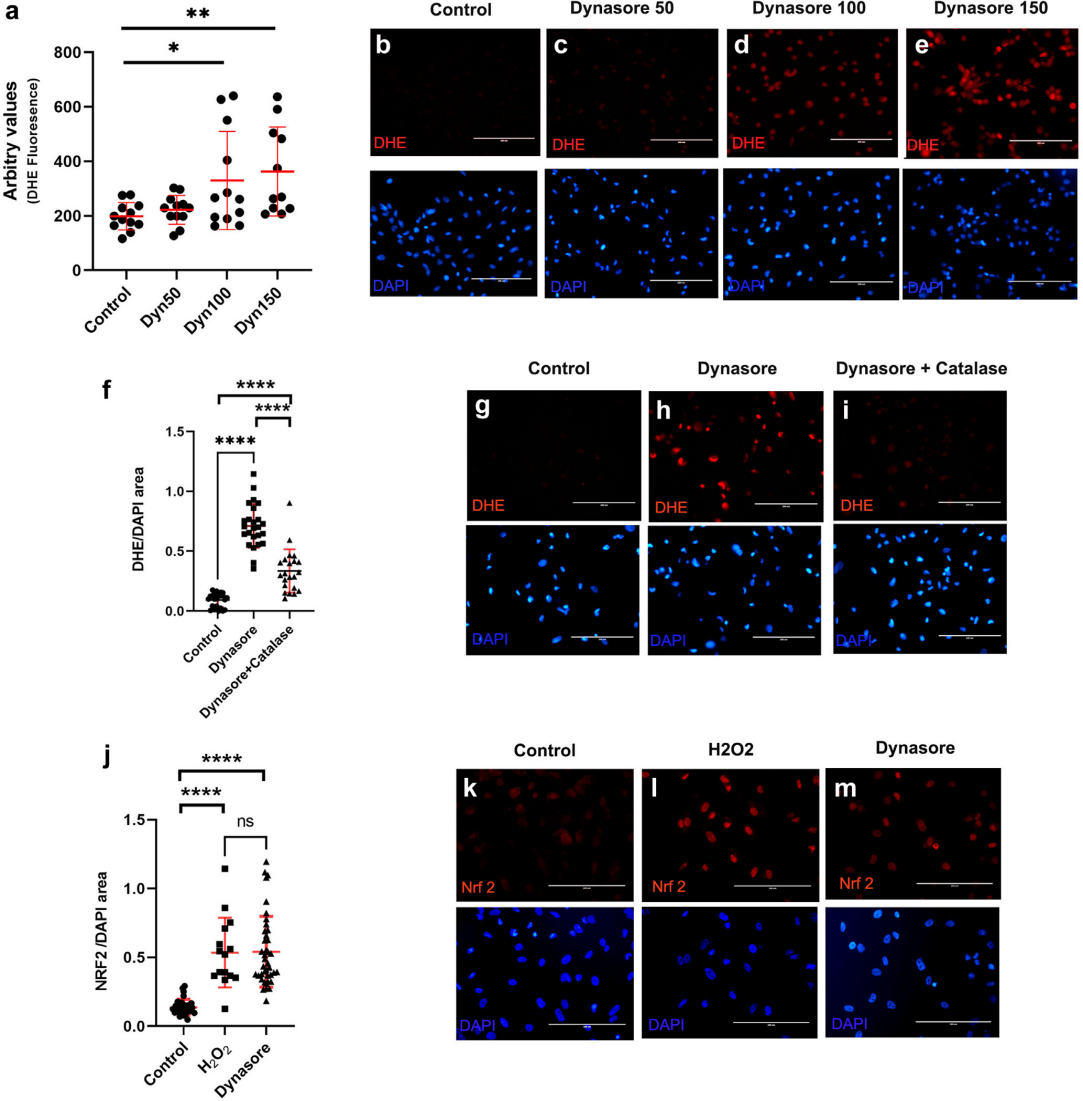
Disruption of endocytosis increases intracellular ROS in cultured human podocytes. (a) Quantification of ROS levels (measured by DHE staining) in human podocytes exposed to increasing amounts of the dynamin inhibitor, dynasore (0, 50, 100, and 150 μM). (b) Image of control podocytes stained with DHE (red). (c–e) Podocytes treated with indicated concentrations of dynasore show dose-dependent increase in nuclear DHE signal. (f) Addition of the antioxidant, catalase (0.5 μM) to dynasore-treated podocytes reduces DHE signal (normalized to nuclear area based on DAPI signal). (g) Control cells stained with DHE. (h) Dynasore treated cell stained with DHE. (i) Cells treated with dynasore and the antioxidant, catalase. (j) Quantification of nuclear Nrf2 levels in podocytes treated with the oxidizing agent H_2_O_2_ or dynasore. (k) Nrf2 levels are low in untreated control podocytes. (l) Treatment of podocytes with the oxidizing agent H_2_O_2_ leads to elevated nuclear Nrf2 staining. (m) Similar increase in nuclear Nrf2 signal was observed following dynasore treatment. DAPI labeling of nuclei in blue. Scale bars = 200microns. ∗*P* < 0.05; ∗∗*P* < 0.01; ∗∗∗∗*P* < 0.0001. DAPI, 4′,6-diamidino-2-phenylindole; DHE, dihydroethidium; Nrf2, nuclear factor erythroid 2–related factor 2; ROS, reactive oxygen species.

To determine if the relationships between endocytosis, oxidative stress, and slit diaphragm integrity that we described in *Drosophila* nephrocytes were recapitulated in human podocytes, we first analyzed the effects of disrupting endocytosis on ROS levels and Nrf2 activity in conditionally immortalized human podocytes. To disrupt endocytosis, we treated cells with dynasore, an inhibitor of dynamin, a protein critical for fission of clathrin-mediated endocytic vesicles from the plasma membrane. Dynasore was previously demonstrated to inhibit endocytosis in cultured podocytes.^49^ Consistent with our findings in fly nephrocytes, disruption of endocytosis increased ROS levels in podocytes, as indicated by increased DHE staining (Figure 4a–e). This effect was reversed by addition of the antioxidant catalase (Figure 4f–i). In agreement with the increased DHE staining following disruption of endocytosis, we found that dynasore treatment led to accumulation of nuclear Nrf2, similar to levels observed when exogenously treated with H_2_O_2_ (Figure 4j–m). Together, these results indicate that disruption of endocytosis leads to increased ROS in cultured human podocytes.

### Inhibiting Endocytosis Disrupts Complex Formation of Nephrin With Other Slit Diaphragm Proteins in A ROS-dependent Manner

**Figure 5:**
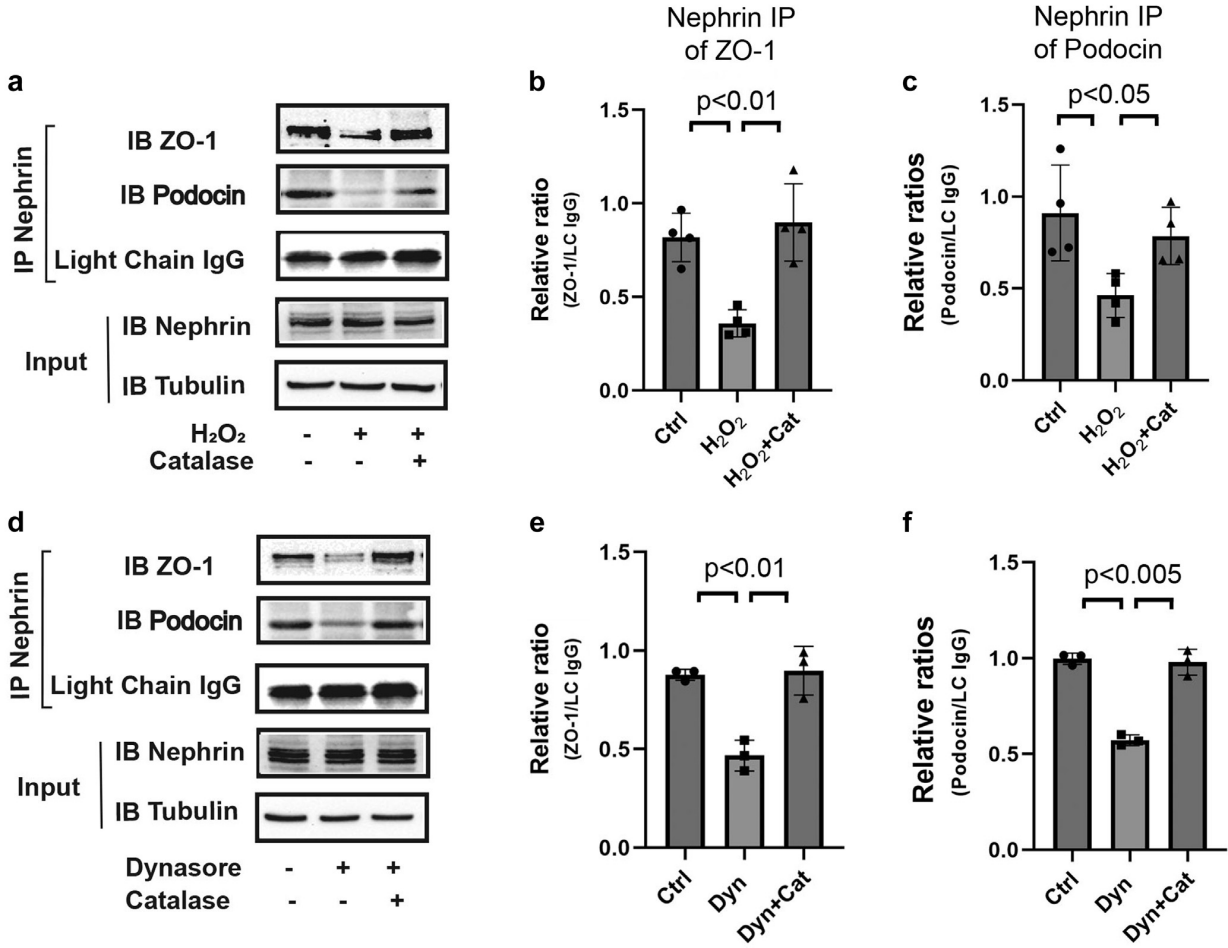
Oxidative stress caused by disruption of endocytosis perturbs slit diaphragm protein interactions. (a) Differentiated podocytes were exposed to H_2_O_2_ (200 μM) in the presence or absence of catalase (0.5 μM) for 48 hours. Cell lysates were immunoprecipitated (IP) with an anti-nephrin antibody followed by immunoblotting (IB) with anti-ZO-1 or anti-podocin antibody. (b, c) Quantification of treatment effects on nephrin association with ZO-1 or podocin. (d) Differentiated podocytes were exposed to dynasore (150 μM, Dyn) in the presence or absence of catalase (0.5 μM) for 48 hours. IP from cell lysates with an anti-nephrin antibody followed by IB with anti-ZO-1 or anti-podocin antibody. The lysates containing the same amount of protein were immunoblotted with anti-nephrin and anti-tubulin antibodies to control protein input. The light chain of immunoglobulin was used as a control for IP. (e, f) Graphs indicating the ratio of densitometry values of each band for the corresponding protein divided by the value of IgG light chain.

Our findings in *Drosophila* nephrocytes indicate that increased ROS caused by disrupting endocytic trafficking can lead to defects in slit diaphragms. Although cultured podocytes do not form slit diaphragm structures, they express core proteins of the slit diaphragm, and those proteins can physically interact with one another. This enables the use of coimmunoprecipitation assays to examine the potential effects of alterations in endocytosis and ROS on the ability of slit diaphragm proteins to complex with one another in these cells. Using lysates from untreated cells, we detected a robust capacity for nephrin to associate with 2 key slit diaphragm proteins, podocin and ZO-1 (see untreated control lanes in Figure 5a; quantified in Figure 5b and c), which are required to maintain slit diaphragm integrity. To test whether nephrin complex formation is altered by cellular ROS levels, we then examined the ability of nephrin to associate with these proteins after treating podocytes with H_2_O_2_. Indeed, ROS treatment alone led to reduced nephrin/podocin or nephrin/ZO-1 complex formation, and as expected, this effect was reversed by addition of the antioxidant catalase (Figure 5a–c).

We then treated podocytes with dynasore to disrupt normal endocytosis. As expected, the association of nephrin with other slit diaphragm proteins was impaired by dynasore treatment (Figure 5d–f), similar to H_2_O_2_ treatment. Importantly, the addition of catalase to dynasore-treated cells was sufficient to restore the ability of these proteins to interact at normal levels (Figure 5d–f), suggesting that elevated ROS caused by disruption of endocytosis is an important contributor to disruption of slit diaphragm protein interactions.

## Discussion

**Figure 6:**
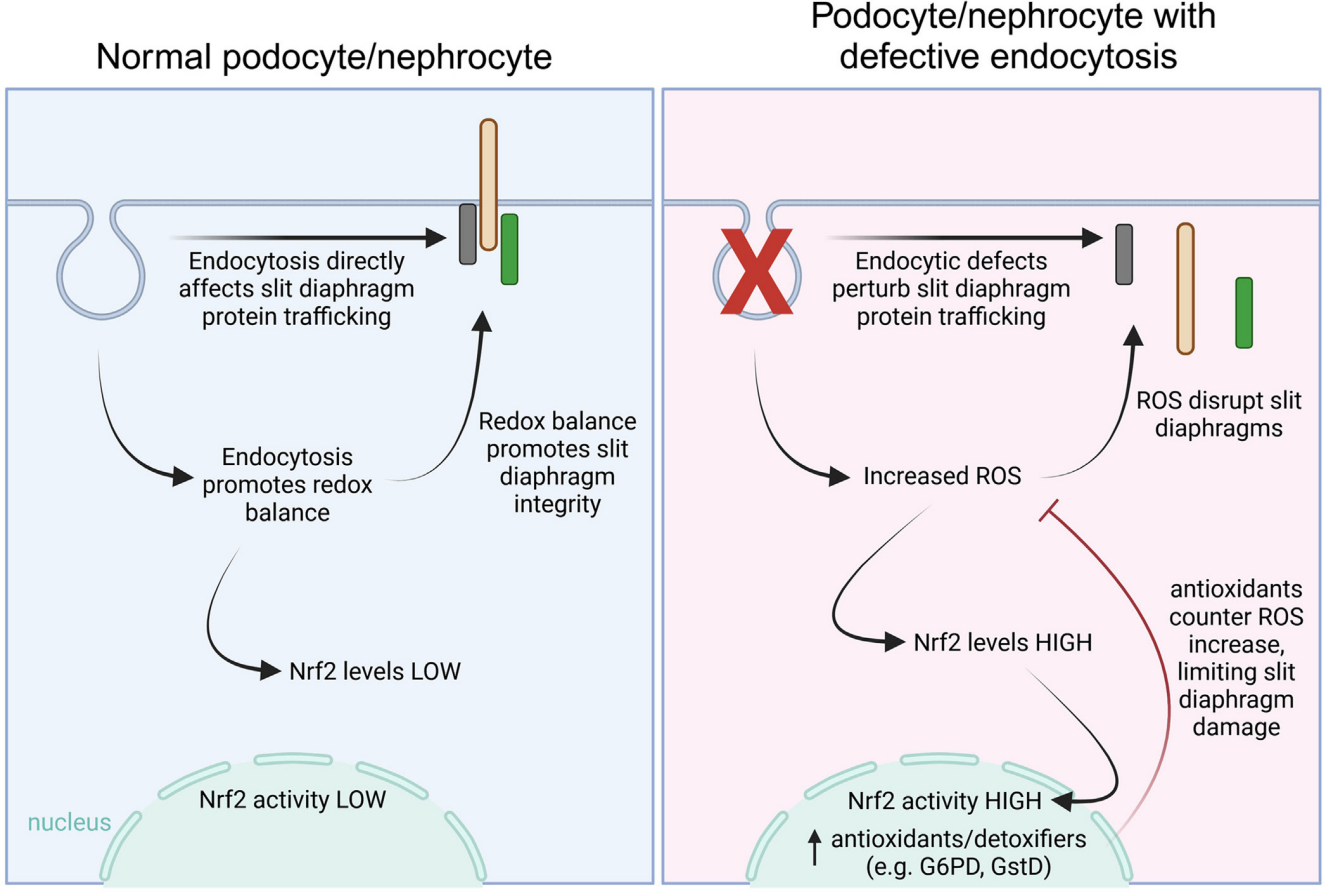
Model depicting key relationships between endocytosis, oxidative stress, Nrf2 signaling, and slit diaphragm dynamics. In normal podocytes or nephrocytes (left panel), endocytosis is known to play a direct role in trafficking slit diaphragm proteins; however, our findings suggest it also contributes to redox homeostasis, which is important for maintaining slit diaphragm integrity. Redox balance also keeps Nrf2 levels and activity low in these cells. However, in cells with defects in endocytosis (right panel), in addition to the previously described direct effects on slit diaphragm protein trafficking, our study suggests that endocytic defects increase ROS levels. Through an unknown mechanism, elevated ROS levels can disrupt slit diaphragm protein localization and interactions, leading to loss of slit diaphragm integrity. Increased ROS levels also promote accumulation of Nrf2 protein, which drives Nrf2 target gene expression, including antioxidant and detoxifying enzymes such as G6PD and GstD. The increased antioxidants buffer ROS levels, which limits the degree of slit diaphragm perturbation. G6PD, glucose-6-phosphate dehydrogenase; Nrf2, nuclear factor erythroid 2–related factor 2; ROS, reactive oxygen species.

Numerous studies demonstrate the importance of proper endocytic trafficking in the context of slit diaphragm integrity, and additional studies have begun to unravel the mechanisms regulating endocytosis of slit diaphragm proteins, such as nephrin.^4^, ^5^, ^6^, ^7^, ^8^, ^9^^,^^13^^,^^50^^,^^51^ However, we still have much to learn regarding these complex events. In the current study, we found that, in addition to its role in trafficking of slit diaphragm proteins, normal endocytosis appears important for maintaining redox balance in *Drosophila* nephrocytes and cultured human podocytes. Furthermore, we found that disruption of endocytosis elicits a conserved antioxidant-promoting response mediated by the Nrf2 pathway, which helps protect slit diaphragms from further damage. Notably, overexpression of key Nrf2 antioxidant-promoting targets, G6PD or catalase, conferred significant additional protection against the detrimental effects of defective endocytosis. Similarly, we found that the addition of catalase to cultured podocytes with impaired endocytosis was able to restore complex formation of major slit diaphragm proteins. Together, our findings reveal oxidative stress as an additional mechanism by which aberrant endocytosis can perturb slit diaphragm integrity (Figure 6).

Our findings also highlight 2 key questions. First, how do defects in endocytosis lead to oxidative stress? There are limited previous data, in any cell type, describing oxidative stress as a consequence of endocytic defects. However, a recent study found a potentially causative mutation in *clavesin-1* in a case of familial childhood steroid-sensitive nephrotic syndrome.^49^ Mechanistically, they found that knockdown of *clavesin-1* in cultured human podocytes disrupts clathrin-mediated endocytosis, which results in increased oxidative stress. *Clavesin-1* is a receptor for a transporter of α-tocopherol (Vitamin E), a known antioxidant. Therefore, it was proposed that loss of *clavesin-1* reduces α-tocopherol levels, resulting in oxidative stress. Further research will be required to determine the various mechanisms by which endocytic defects may lead to oxidative stress in podocytes.

The second major question to be resolved is how oxidative stress leads to disruption of slit diaphragms. Although numerous studies indicate important connections between oxidative stress and alterations in slit diaphragm integrity, we know little about the underlying mechanisms. Oxidative stress can directly or indirectly affect virtually every aspect of cell function and behavior; thus it will require extensive studies to explore the many possibilities. It is tempting to speculate that slit diaphragm proteins, such as nephrin, may be directly oxidized by ROS. Protein oxidation damages the protein, which can lead to its degradation^52^; and reduced nephrin levels have been reported in various forms of kidney disease. Intriguingly, a previous study in rats found that high fat diet led to renal dysfunction with reduced protein levels of nephrin and podocin, and supplementing the high fat diet with the antioxidant resveratrol significantly restored the levels of both proteins.^53^ Another possible mechanism stems from the observation that the activity of some kinases and phosphatases are ROS-sensitive.^54^ Indeed, previous research suggests nephrin's phosphorylation state changes in response to changes in ROS levels,^55^ which can affect its interaction with other proteins of the slit diaphragm.^3^ These observations, in conjunction with the known importance of nephrin phosphorylation on its function, trafficking, and turnover, suggest a model in which increased ROS levels affect nephrin phosphorylation, and thus its cellular dynamics. Hopefully, future research will be able to test these hypotheses. Improving our understanding of these complex relationships will help identify the forms of kidney disease, or even specific patients, who may benefit from relevant therapeutic options, such as antioxidant treatments or Nrf2 activators (i.e., bardoxolone methyl).

## Disclosure

All the authors declared no competing interests.

## Supplementary Material

Supplementary File (PDF)

**Figure S1.** Systemic upregulation of the Nrf2 reporter GstD>GFP by bardoxolone methyl in flies, and DHE staining in Amn and Cubn depleted nephrocytes.

## References

[1] Ruotsalainen V., Ljungberg P., Wartiovaara J. (1999). Nephrin is specifically located at the slit diaphragm of glomerular podocytes. Proc Natl Acad Sci U S A.

[2] Barletta G.M., Kovari I.A., Verma R.K., Kerjaschki D., Holzman L.B. (2003). Nephrin and Neph1 co-localize at the podocyte foot process intercellular junction and form cis hetero-oligomers. J Biol Chem.

[3] Martin C.E., Jones N. (2018). Nephrin signaling in the podocyte: an updated view of signal regulation at the slit diaphragm and beyond. Front Endocrinol (Lausanne).

[4] Quack I., Rump L.C., Gerke P. (2006). beta-Arrestin2 mediates nephrin endocytosis and impairs slit diaphragm integrity. Proc Natl Acad Sci U S A.

[5] Quack I., Woznowski M., Potthoff S.A. (2011). PKC alpha mediates beta-arrestin2-dependent nephrin endocytosis in hyperglycemia. J Biol Chem.

[6] Teng B., Schroder P., Müller-Deile J. (2016). CIN85 deficiency prevents nephrin endocytosis and proteinuria in diabetes. Diabetes.

[7] Martin C.E., New L.A., Phippen N.J. (2020). Multivalent nephrin-Nck interactions define a threshold for clustering and tyrosine-dependent nephrin endocytosis. J Cell Sci.

[8] Tossidou I., Teng B., Menne J. (2010). Podocytic PKC-alpha is regulated in murine and human diabetes and mediates nephrin endocytosis. PLoS One.

[9] Qin X.S., Tsukaguchi H., Shono A., Yamamoto A., Kurihara H., Doi T. (2009). Phosphorylation of nephrin triggers its internalization by raft-mediated endocytosis. J Am Soc Nephrol.

[10] Soda K., Balkin D.M., Ferguson S.M. (2012). Role of dynamin, synaptojanin, and endophilin in podocyte foot processes. J Clin Invest.

[11] Tian X., Bunda P., Ishibe S. (2022). Podocyte endocytosis in regulating the glomerular filtration barrier. Front Med (Lausanne).

[12] Mysh M., Poulton J.S. (2021). The basolateral polarity module promotes slit diaphragm formation in *Drosophila* nephrocytes, a model of vertebrate podocytes. J Am Soc Nephrol.

[13] Lang K., Milosavljevic J., Heinkele H. (2022). Selective endocytosis controls slit diaphragm maintenance and dynamics in *Drosophila* nephrocytes. eLife.

[14] Fu Y., Zhu J.Y., Zhang F., Richman A., Zhao Z., Han Z. (2017). Comprehensive functional analysis of Rab GTPases in *Drosophila* nephrocytes. Cell Tissue Res.

[15] Wang L., Wen P., van de Leemput J., Zhao Z., Han Z. (2021). Slit diaphragm maintenance requires dynamic clathrin-mediated endocytosis facilitated by AP-2, Lap, Aux and Hsc70-4 in nephrocytes. Cell Biosci.

[16] Cui Y W.Y., Luo M., Miao L., Cui W. (2018). Emerging roles of nuclear factor erythroid 2 related Factor 2 in podocytopathy: a review. J Clin Exp Nephrol.

[17] Daenen K., Andries A., Mekahli D., Van Schepdael A., Jouret F., Bammens B. (2019). Oxidative stress in chronic kidney disease. Pediatr Nephrol.

[18] Ratliff B.B., Abdulmahdi W., Pawar R., Wolin M.S. (2016). Oxidant mechanisms in renal injury and disease. Antioxid Redox Signal.

[19] Sureshbabu A., Ryter S.W., Choi M.E. (2015). Oxidative stress and autophagy: crucial modulators of kidney injury. Redox Biol.

[20] Tang J., Yan H., Zhuang S. (2012). Inflammation and oxidative stress in obesity-related glomerulopathy. Int J Nephrol.

[21] Shibata S., Nagase M., Yoshida S., Kawachi H., Fujita T. (2007). Podocyte as the target for aldosterone: roles of oxidative stress and Sgk1. Hypertension.

[22] Gwinner W., Landmesser U., Brandes R.P. (1997). Reactive oxygen species and antioxidant defense in puromycin aminonucleoside glomerulopathy. J Am Soc Nephrol.

[23] Jha J.C., Thallas-Bonke V., Banal C. (2016). Podocyte-specific Nox4 deletion affords renoprotection in a mouse model of diabetic nephropathy. Diabetologia.

[24] Su M., Dhoopun A.R., Yuan Y. (2013). Mitochondrial dysfunction is an early event in aldosterone-induced podocyte injury. Am J Physiol Ren Physiol.

[25] Szeto H.H., Liu S., Soong Y. (2017). Mitochondria protection after acute ischemia prevents prolonged upregulation of IL-1β and IL-18 and arrests CKD. J Am Soc Nephrol.

[26] Diomedi-Camassei F., Di Giandomenico S., Santorelli F.M. (2007). COQ2 nephropathy: a newly described inherited mitochondriopathy with primary renal involvement. J Am Soc Nephrol.

[27] Heeringa S.F., Chernin G., Chaki M. (2011). COQ6 mutations in human patients produce nephrotic syndrome with sensorineural deafness. J Clin Invest.

[28] Hermle T., Braun D.A., Helmstädter M., Huber T.B., Hildebrandt F. (2017). Modeling monogenic human nephrotic syndrome in the *Drosophila* garland cell nephrocyte. J Am Soc Nephrol.

[29] Zhu J.Y., Fu Y., Richman A., Zhao Z., Ray P.E., Han Z. (2017). A personalized model of COQ2 nephropathy rescued by the wild-type COQ2 allele or dietary coenzyme Q10 supplementation. J Am Soc Nephrol.

[30] Mitsuishi Y., Taguchi K., Kawatani Y. (2012). Nrf2 redirects glucose and glutamine into anabolic pathways in metabolic reprogramming. Cancer Cell.

[31] Lee J.M., Calkins M.J., Chan K., Kan Y.W., Johnson J.A. (2003). Identification of the NF-E2-related factor-2-dependent genes conferring protection against oxidative stress in primary cortical astrocytes using oligonucleotide microarray analysis. J Biol Chem.

[32] Sykiotis G.P., Bohmann D. (2008). Keap1/Nrf2 signaling regulates oxidative stress tolerance and lifespan in *Drosophila*. Dev Cell.

[33] Saleem M.A., O’Hare M.J., Reiser J. (2002). A conditionally immortalized human podocyte cell line demonstrating nephrin and podocin expression. J Am Soc Nephrol.

[34] Troha K., Nagy P., Pivovar A., Lazzaro B.P., Hartley P.S., Buchon N. (2019). Nephrocytes remove microbiota-derived peptidoglycan from systemic circulation to maintain immune homeostasis. Immunity.

[35] Weavers H., Prieto-Sánchez S., Grawe F. (2009). The insect nephrocyte is a podocyte-like cell with a filtration slit diaphragm. Nature.

[36] Zhuang S., Shao H., Guo F., Trimble R., Pearce E., Abmayr S.M. (2009). Sns and Kirre, the *Drosophila* orthologs of nephrin and Neph1, direct adhesion, fusion and formation of a slit diaphragm-like structure in insect nephrocytes. Development.

[37] Heiden S., Siwek R., Lotz M.L. (2021). Apical-basal polarity regulators are essential for slit diaphragm assembly and endocytosis in *Drosophila* nephrocytes. Cell Mol Life Sci.

[38] Feng X., Hong X., Fan Q. (2021). dCubilin- or dAMN-mediated protein reabsorption in *Drosophila* nephrocytes modulates longevity. Dis Model Mech.

[39] Atienza-Manuel A., Castillo-Mancho V., De Renzis S., Culi J., Ruiz-Gómez M. (2021). Endocytosis mediated by an atypical CUBAM complex modulates slit diaphragm dynamics in nephrocytes. Development.

[40] Zhang F., Zhao Y., Chao Y., Muir K., Han Z. (2013). Cubilin and amnionless mediate protein reabsorption in *Drosophila* nephrocytes. J Am Soc Nephrol.

[41] Odenthal J., Dittrich S., Ludwig V. (2023). Modeling of ACTN4-based podocytopathy using *Drosophila* nephrocytes. Kidney Int Rep.

[42] Carrasco-Rando M., Prieto-Sánchez S., Culi J., Tutor A.S., Ruiz-Gómez M. (2019). A specific isoform of Pyd/ZO-1 mediates junctional remodeling and formation of slit diaphragms. J Cell Biol.

[43] Spitz D., Comas M., Gerstner L. (2022). mTOR-dependent autophagy regulates slit diaphragm density in podocyte-like *Drosophila* nephrocytes. Cells.

[44] Bindokas V.P., Jordán J., Lee C.C., Miller R.J. (1996). Superoxide production in rat hippocampal neurons: selective imaging with hydroethidine. J Neurosci.

[45] Tonelli C., Chio I.I.C., Tuveson D.A. (2018). Transcriptional regulation by Nrf2. Antioxid Redox Signal.

[46] Legan S.K., Rebrin I., Mockett R.J. (2008). Overexpression of glucose-6-phosphate dehydrogenase extends the life span of *Drosophila melanogaster*. J Biol Chem.

[47] Stanton R.C. (2012). Glucose-6-phosphate dehydrogenase, NADPH, and cell survival. IUBMB Life.

[48] Hanschmann E.M., Godoy J.R., Berndt C., Hudemann C., Lillig C.H. (2013). Thioredoxins, glutaredoxins, and peroxiredoxins--molecular mechanisms and health significance: from cofactors to antioxidants to redox signaling. Antioxid Redox Signal.

[49] Lane B.M., Chryst-Stangl M., Wu G. (2022). Steroid-sensitive nephrotic syndrome candidate gene CLVS1 regulates podocyte oxidative stress and endocytosis. JCI Insight.

[50] Dumont V., Tolvanen T.A., Kuusela S. (2017). PACSIN2 accelerates nephrin trafficking and is up-regulated in diabetic kidney disease. FASEB J.

[51] Tossidou I., Himmelseher E., Teng B., Haller H., Schiffer M. (2014). SUMOylation determines turnover and localization of nephrin at the plasma membrane. Kidney Int.

[52] Hohn A., Konig J., Grune T. (2013). Protein oxidation in aging and the removal of oxidized proteins. J Proteomics.

[53] Pan Q.R., Ren Y.L., Zhu J.J. (2014). Resveratrol increases nephrin and podocin expression and alleviates renal damage in rats fed a high-fat diet. Nutrients.

[54] Bogeski I., Bozem M., Sternfeld L., Hofer H.W., Schulz I. (2006). Inhibition of protein tyrosine phosphatase 1B by reactive oxygen species leads to maintenance of Ca2+ influx following store depletion in HEK 293 cells. Cell Calcium.

[55] Tian Y., Guo H., Miao X. (2020). Nestin protects podocyte from injury in lupus nephritis by mitophagy and oxidative stress. Cell Death Dis.

