# Supplementary material for: Oxidative Stress Contributes to Slit Diaphragm Defects Caused by Disruption of Endocytosis
Source: Kidney Int Rep. 2023 Nov 25;9(2):451–63. doi: 10.1016/j.ekir.2023.11.018 (PMC10851022; doi:10.1016/j.ekir.2023.11.018)
Supplement: Supplementary File (PDF) [file mmc1.pdf]

Supplemental Figure 1

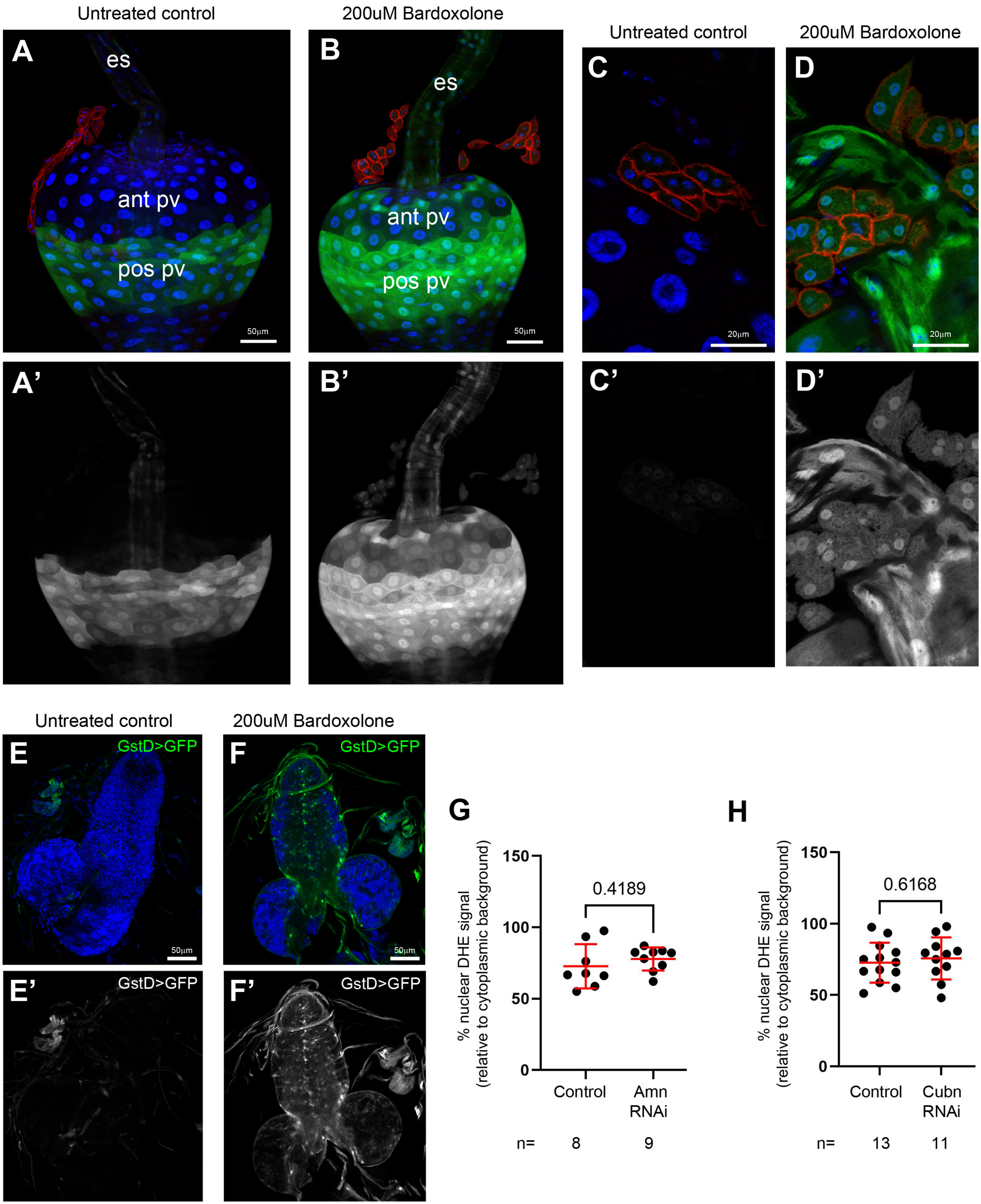

Supplemental Figure S1. Systemic upregulation of the Nrf2 reporter GstD>GFP by bardoxolone methyl in flies, and DHE staining in Amn and Cubn depleted nephrocytes.

A. Untreated control larvae expressing the Nrf2 reporter GstD>GFP have low GFP expression in nephrocytes, the esophagus (es), and anterior cells of the proventriculus (ant pv), but high levels of expression in the posterior proventricular cells (pos pv); the nephrocytes are easily distinguished by the high levels of ZO-1 (red). DAPI staining of nuclei in blue. A'. The GFP channel alone shown in gray scale. B-B'. GstD>GFP larvae reared on food supplemented with 200μM Bardoxolone express significantly higher levels of GFP in all cell types. C-D'. Higher magnification images indicate strong upregulation of GstD>GFP in the nephrocytes (ZO-1 positive) of bardoxolone treated animals. E-F'. Similar degree of upregulation can be observed in the neurons and other cells of the larval brain. G. Quantification of DHE staining in control and Amn RNAi nephrocytes. H. Quantification of DHE in Cubn RNAi nephrocytes. Neither is significantly different from respective controls based on Welch's unpaired t-test.
